# Clinician decision-making in non-functioning pituitary adenomas: an Australian and New Zealand interdisciplinary survey study

**DOI:** 10.1007/s11102-026-01716-3

**Published:** 2026-07-03

**Authors:** Edward Mignone, Frank Saran, Alkis Psaltis, Alistair Jukes, Ryan Paul, Richard W Carroll, Peter Gorayski, Lauren Cooper, Ian Chapman, David J Torpy, Sunita MC De Sousa

**Affiliations:** 1 School of Medicine, Adelaide University, Adelaide, SA Australia; 2 Department of Endocrinology, Royal Adelaide Hospital, Adelaide, Australia; 3 Department of Radiation Oncology, Auckland City Hospital, Auckland, Australia; 4 Department of Radiation Oncology, Royal Adelaide Hospital, Adelaide, Australia; 5 Department of Otolaryngology, Queen Elizabeth Hospital, Adelaide, Australia; 6 Department of Neurosurgery, Royal Adelaide Hospital, Adelaide, Australia; 7 Department of Endocrinology, Waikato Hospital, Waikato, New Zealand; 8 Department of Endocrinology, Capital and Coast District Health Board, Wellington, New Zealand; 9 Department of Otolaryngology, Flinders Medical Centre, Adelaide, Australia

**Keywords:** Non-functioning pituitary adenoma, Pituitary incidentaloma, Clinical practice variation, Multidisciplinary team

## Abstract

**Purpose:**

Non-functioning pituitary adenomas (NFPA) are one of the most frequent pituitary adenoma subtypes, yet dedicated management guidelines do not exist. This study aimed to characterise real-world clinical decision-making across the spectrum of NFPA presentations and to identify areas of practice conformity versus heterogeneity within and between specialty groups.

**Methods:**

A cross-sectional, anonymous electronic survey was administered to endocrinologists, neurosurgeons, ENT surgeons, and radiation oncologists involved in pituitary adenoma care in Australia and New Zealand between January and April 2025. Three clinical scenarios were presented: an incidental microadenoma, an incidental asymptomatic macroadenoma, and a symptomatic macroadenoma with visual compromise. Concordance was defined as greater than 70% agreement on a single response within any group.

**Results:**

A total of 145 clinicians completed the survey (99 endocrinologists, 31 surgeons, 15 radiation oncologists). Areas of conformity included broad anterior pituitary hormonal evaluation, conservative management of incidental asymptomatic macroadenomas, and active intervention for symptomatic disease with visual loss. Significant heterogeneity was identified in ophthalmological assessment, repeat imaging intervals, post-operative MRI and visual field timing, and multidisciplinary team (MDT) meeting referral rates. Compared to general endocrinologists, pituitary subspecialty endocrinologists were significantly more likely to refer patients for MDT discussion and know the caseload of the surgeon to whom they were referring, and less likely to measure serum cortisol immediately post-operatively.

**Conclusion:**

This survey identifies clinically important variation in NFPA management, concentrated in domains lacking guideline recommendations. These findings highlight the need for dedicated NFPA guidelines spanning the full spectrum of NFPA presentations.

**Supplementary Information:**

The online version contains supplementary material available at https://doi.org/10.1007/s11102-026-01716-3.

## Introduction

Pituitary adenomas (PA) are benign neoplasms arising from clonal expansion of anterior pituitary cells [1]. Non-functioning pituitary adenomas (NFPA) are one of the most common subtypes, comprising 14–54% of all PAs with a prevalence of up to 41.3 per 100,000 persons [2]. Historically, NFPAs most commonly presented with symptoms of mass effect including headache, visual compromise or symptoms of hypopituitarism [3]. With increasing rates of neuroimaging performed for unrelated indications and advances in imaging techniques, incidental detection is now an increasingly common presentation [4]. Despite representing one of the most frequently encountered PA subtypes, NFPAs remain without a comprehensive, dedicated management guideline, contrasting against the international consensus guidelines in place for prolactinoma, acromegaly and Cushing’s disease [5–10]. The only existing evidence-based guidelines specifically addressing symptomatic NFPA management were published in 2016 by the Congress of Neurological Surgeons and the American Association of Neurological Surgeons [11], which are predominantly neurosurgical in scope with many aspects of endocrinological and multidisciplinary post-operative management either not addressed or supported only by low-level evidence.

There is no consensus on optimal pre-, peri-, and post-operative management of NFPA, including the timing, frequency, and duration of endocrine, radiological, and ophthalmological assessments, and substantial variations in clinical practice have been reported [12]. Most recently, the 2025 Pituitary Society international consensus guideline on pituitary incidentalomas has provided guidance for the assessment and surveillance of incidentally discovered pituitary lesions, which includes NFPAs [13]. However, real-world practice can diverge from these recommendations for multiple reasons including resource availability, awareness, and variation by subspecialty expertise. Furthermore, by design this guideline addresses only incidentally discovered lesions and does not provide recommendations for symptomatic NFPA presentations, which remain the most clinically urgent and complex management scenario. Importantly, the present survey was conducted between January and April 2025, prior to online publication of the Pituitary Society incidentaloma guidelines in June 2025. These guidelines are therefore referenced here as a post-hoc comparator to contextualise observed practice patterns against contemporaneous recommendations, rather than to assess prospective guideline adherence.

Against this background, we conducted a survey of endocrinologists, neurosurgeons, ENT surgeons, and radiation oncologists with experience in managing pituitary tumours across Australia and New Zealand. The survey aimed to characterise real-world clinical decision-making across three clinical scenarios spanning the spectrum of NFPA presentations and to describe the spectrum of contemporaneous practice within specialty groups.

## Methods

We performed a cross-sectional, anonymous electronic survey of individual clinicians involved in the management of NFPAs across Australia and New Zealand (ANZ). The survey was administered between January to April 2025. Eligible participants included practising endocrinologists, neurosurgeons, ear, nose and throat (ENT) surgeons and radiation oncologists involved in pituitary tumour care. The survey instrument was constructed using Microsoft Teams Forms (Microsoft Corporation, Redmond, WA, USA). The question set (Supplementary Material) was developed collaboratively by the South Australia Pituitary Research Group, a multidisciplinary pituitary team comprising endocrinologists, neurosurgeons, ENT surgeons and radiation oncologists with experience in pituitary tumour management. The question set was divided into the three commonest clinical scenarios in NFPA management: an incidental intrasellar microadenoma, an incidental asymptomatic intrasellar macroadenoma, and a symptomatic presentation of a macroadenoma with visual deficits. An additional scenario was provided to radiation oncologists to elicit their irradiation practices. The survey was disseminated to members of relevant specialty societies – the Endocrine Society of Australia, New Zealand Endocrine Society, Neurosurgical Society of Australia, and the Australia and New Zealand Rhinologic Society. Radiation oncologist answers were limited to questions regarding radiotherapy management to match their scope of practice. No identifiable personal data were obtained. Ethical approval was obtained through the Central Adelaide Local Health Network (20514).

For analysis, data were exported directly from Microsoft Teams into a spreadsheet format. Neurosurgeons and ENT surgeons were combined into a single surgeon group given the shared surgical scope of their pituitary practice and the limited sample size of each individual surgical specialty. Endocrinologists were subclassified into two subgroups for secondary analysis. Pituitary subspecialty endocrinologists (PSE) were defined as those who reported seeing five or more pituitary cases per month and either working within a pituitary multidisciplinary team (MDT) clinic or attending a hospital-based pituitary MDT meeting at least monthly. The remaining endocrinologists were classified as general endocrinologists (GE). This definition was selected to identify endocrinologists with both institutional MDT integration and sufficient personal case volume to reflect subspecialty-level pituitary practice. Respondents were blinded to this categorisation. Statistical analyses were performed using IBM SPSS Statistics (version 29.0, IBM Corp., Armonk, NY), noting that statistical analysis was not possible for the radiation oncologist responses to radiotherapy-specific questions due to small sample sizes. Between-group comparisons were performed using the Fisher’s exact test for binary variables and Mann-Whitney U test for ordinal variables. Responses were also analysed to determine areas of concordance and variability, with concordance defined as > 70% of respondents agreeing on a single response as a whole group, or in any single specialty group. Areas of variability were defined by a lack of concordance, or if there was a statistically significant difference between two groups.

## Results

### Participant demographics

A total of 145 clinicians (121 (83%) from Australia, 24 (17%) from New Zealand) completed the survey, comprising 99 endocrinologists (68%), 17 neurosurgeons (12%), 14 ENT surgeons (10%), and 15 radiation oncologists (10%) (Table 1). Practice location was predominantly metropolitan (89%), with 11% from regional and remote areas. Of the total study group, 69 participants attended a pituitary MDT meeting at least monthly (48%), and 28 worked within a pituitary MDT clinic held at least monthly (19%). Most surgeons (68%) performed over 20 pituitary operations per year, whilst a majority of endocrinologists (58%) saw five or more pituitary cases per month (Table 1). Within the endocrinologist group, 29 endocrinologists met the PSE criteria for the purpose of this study (29%), compared to 70 endocrinologists who were classified as GE (71%).

**Table 1:** Baseline demographics of respondents

	Endocrinologists	Neurosurgeons	ENT surgeons	Radiation oncologists	Total (%)
Total respondents	99	17	14	15	145 (100%)
Metropolitan practice	85	16	14	14	129 (89%)
Regional/Remote practice	14	1	0	1	16 (11%)
Attends monthly MDT	39	11	10	9	69 (48%)
Works at a monthly MDT	17	5	5	1	28 (19%)
Low volume case exposure^*^	57	5	5	13	80 (55%)
Moderate volume case exposure^**^	N/A	9	7	N/A	16 (11%)
High volume case exposure^***^	42	3	2	2	49 (34%)

* per month volume of < 5 pituitary cases per month for endocrinologists, < 20 pituitary cases per year for surgeons and radiation oncologists

** per month volume of 21-50 pituitary surgeries per year for surgeons

*** per month volume of ≥ 5 pituitary cases per month for endocrinologists, >50 cases per year for surgeons and ≥ 20 cases per year for radiation oncologists

*Abbreviations: MDT*, multi-disciplinary team; *ENT*, Ear, Nose and Throat

### Clinical scenario 1: incidental intrasellar non-functioning microadenoma

The first scenario stem presented a 30-year-old man presenting with sport-related head trauma leading to neuroimaging which detected an incidental 6 mm intrasellar pituitary adenoma with no visual field deficits on examination (Supplementary Material). Endocrinologists were more likely than surgeons to request a complete pituitary hormonal evaluation, including cortisol (97% vs. 61%, *p* < 0.0001), thyroid stimulating hormone (TSH) (95% vs. 65%, *p* < 0.0001), free T4 (100% vs. 52%, *p* < 0.0001), follicle stimulating hormone (FSH) (90% vs. 58%, *p* = 0.0003), luteinizing hormone (LH) (93% vs. 52%, *p* < 0.0001), oestrogen for women (73% vs. 32%, *p* < 0.0001), androgens for men (90% vs. 45%, *p* < 0.0001), prolactin (100% vs. 65%, *p* < 0.0001), and insulin-like growth factor-1 (IGF-1) (98% vs. 58%, *p* < 0.0001).

**Fig. 1:**
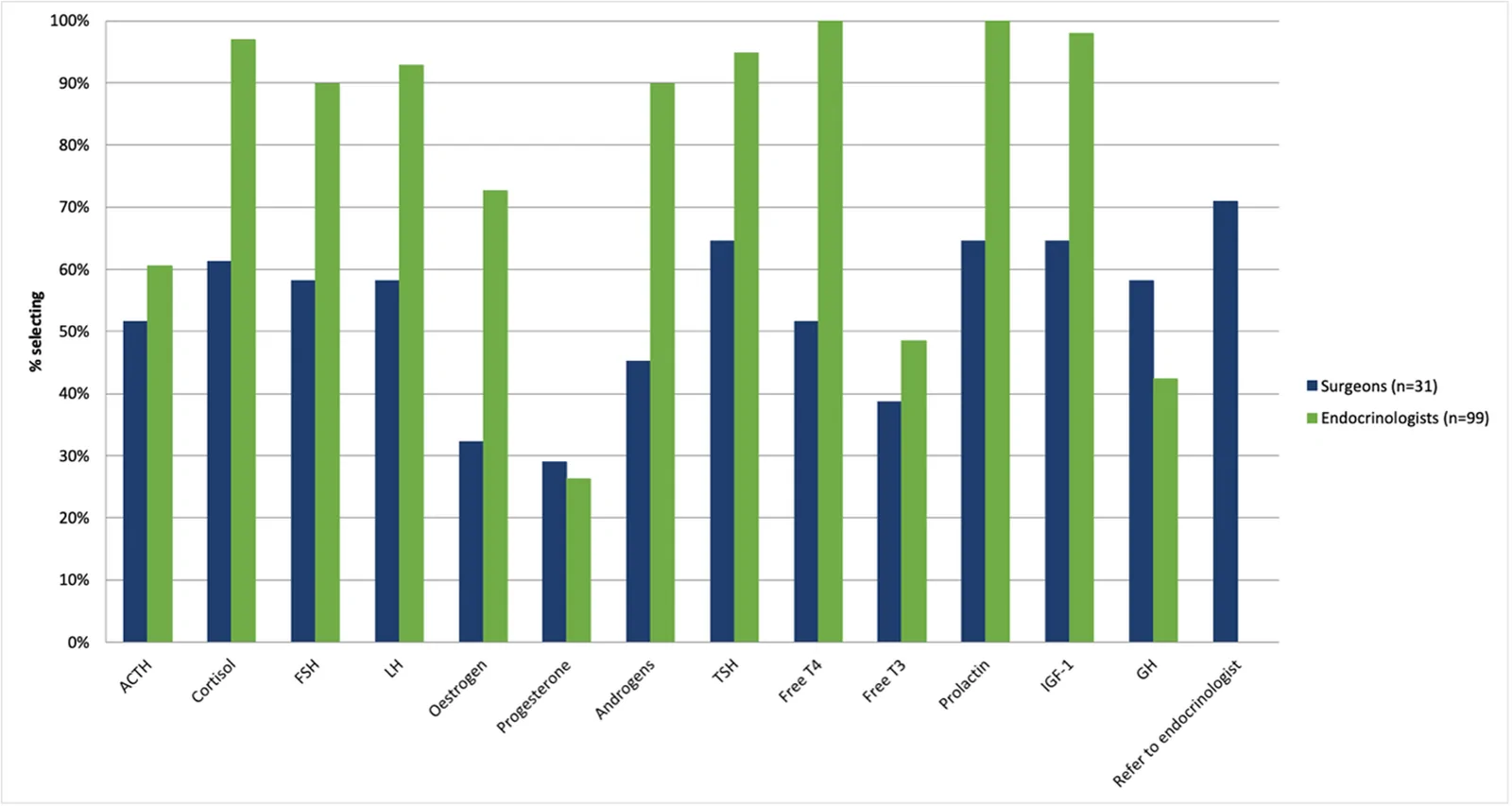
Hormonal evaluation selected for an incidental intrasellar non-functioning microadenoma by surgeons and endocrinologists. p<0.0001 (Fisher's exact test) for cortisol, FSH, LH, oestrogen (for females), androgens (for males), TSH, free T4, prolactin, IGF-1. Fisher's exact test. Abbreviations: *ACTH*, Adrenocorticotropic hormone; *FSH*, Follicle-Stimulating Hormone; *LH*, Luteinizing hormone; *TSH*, Thyroid stimulating hormone; *IGF-1*, Insulin-like growth factor 1; *GH*, Growth hormone

Ophthalmological evaluation differed significantly between surgeons and endocrinologists (*p* < 0.0001) (Fig. 1). The majority of surgeons arranged formal ophthalmological assessment (19/31, 61%), with 74% of this group selecting combined visual field perimetry and optical coherence tomography (OCT). In contrast, the majority of endocrinologists arranged no formal ophthalmological assessment (78/99, 79%), and of those who did, 90% selected visual field perimetry alone. The most common timeframe for repeat MRI evaluation was 12 months (61% of surgeons and 78% of endocrinologists). Timing of repeat MRI differed significantly between surgeons and endocrinologists (*p* = 0.003), with surgeons more likely to arrange shorter interval imaging at 3–6 months (39% vs. 14%) and a small proportion of endocrinologists deferring imaging to 2 + years (7% vs. 0%). Noting that the initial imaging did not include gadolinium, the majority of respondents included gadolinium on the repeat study (71% of surgeons, 65% of endocrinologists). Timing of repeat pituitary hormonal evaluation was heterogenous, most commonly arranged at 12 months (39% of surgeons, 55% of endocrinologists), with a higher proportion of surgeons arranging shorter interval evaluation at 3–6 months (32% vs. 16%), and some clinicians performing no repeat hormonal evaluation (19% vs. 17%) (*p* = 0.214).

### Clinical scenario 2: incidental intrasellar non-functioning macroadenoma

Scenario 2 described a 70-year-old man who had neuroimaging performed after a fall, detecting a 15 mm macroadenoma with unilateral Knosp grade 2 cavernous sinus invasion but no suprasellar extension. He was biochemically eupituitary with no visual field defects on examination. Participants were asked to respond with their single next step in management, with 94% of respondents proceeding with a conservative approach rather than surgery or radiotherapy. Surgeons were most likely to arrange repeat evaluation in 6 months (48% of surgeons vs. 37% of endocrinologists), whilst endocrinologists more commonly referred for formal visual field testing (39% vs. 29%); a smaller number organised repeat evaluation in 12 months (23% vs. 15%). Notably, 8% of endocrinologists selected immediate surgical referral compared to no surgeons, despite the absence of visual compromise, hypopituitarism or tumour growth in this scenario. None of these differences reached statistical significance (*p* = 0.611).

### Clinical scenario 3: symptomatic presentation of a non-functioning macroadenoma with visual compromise

**Fig. 2:**
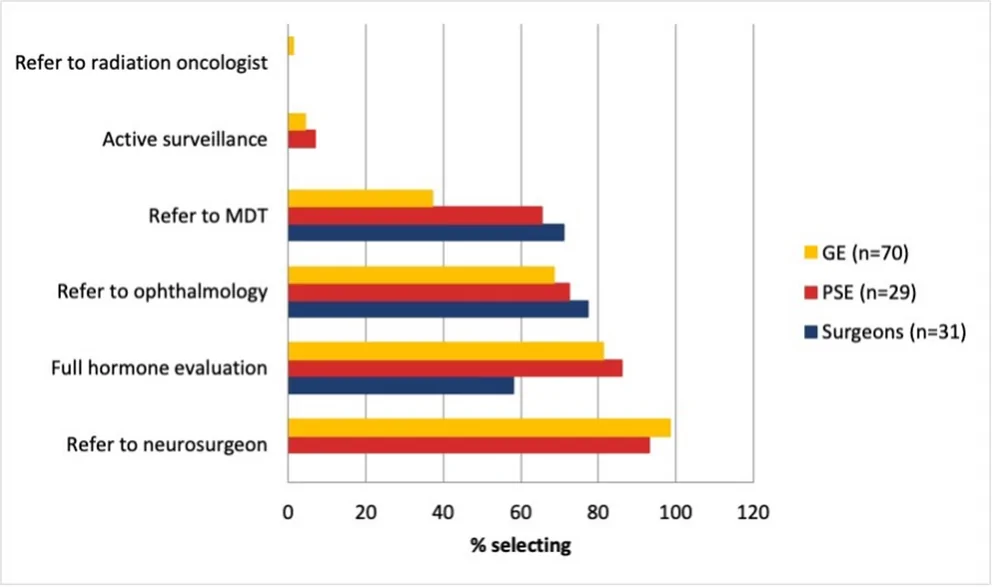
Initial management steps selected for a symptomatic non-functioning pituitary macroadenoma with visual compromise, stratified by specialty group. Responses were multi-select. MDT referral differed significantly between pituitary subspecialty and general endocrinologists (66% vs 37%, p=0.014). Abbreviations: *PSE*, Pituitary subspecialty endocrinologist; *GE*, General endocrinologist; *MDT*, multi-disciplinary team

Respondents were asked to select all initial management steps (responses not mutually exclusive) for a 58-year-old man presenting with superior quadrantanopia, a 19 mm non-functioning macroadenoma with right-sided Knosp grade 2 cavernous sinus invasion and a normal prolactin (Fig. 2). No surgeon or endocrinologists chose active surveillance. The most frequent action among endocrinologists was referral to a neurosurgeon (97%). Pituitary hormone evaluation was selected by a significantly higher proportion of endocrinologists than surgeons (83% vs. 58%, *p* = 0.007). Formal ophthalmology referral was selected by 72% of all respondents with no significant interspecialty difference. MDT referral was selected by 52% of all respondents overall, and was significantly more common among surgeons than endocrinologists (71% vs. 46%, *p* = 0.015). Within the endocrinologist group, PSE were significantly more likely than GE to refer for MDT discussion (66% vs. 37%, *p* = 0.014).

Endocrinologists were asked to indicate their awareness of the annual operative pituitary caseload of the surgeon to whom they most commonly refer. PSE were significantly more likely than GE to be aware of their referring surgeon’s caseload (93% vs. 51%, *p* = 0.005).

**Fig. 3:**
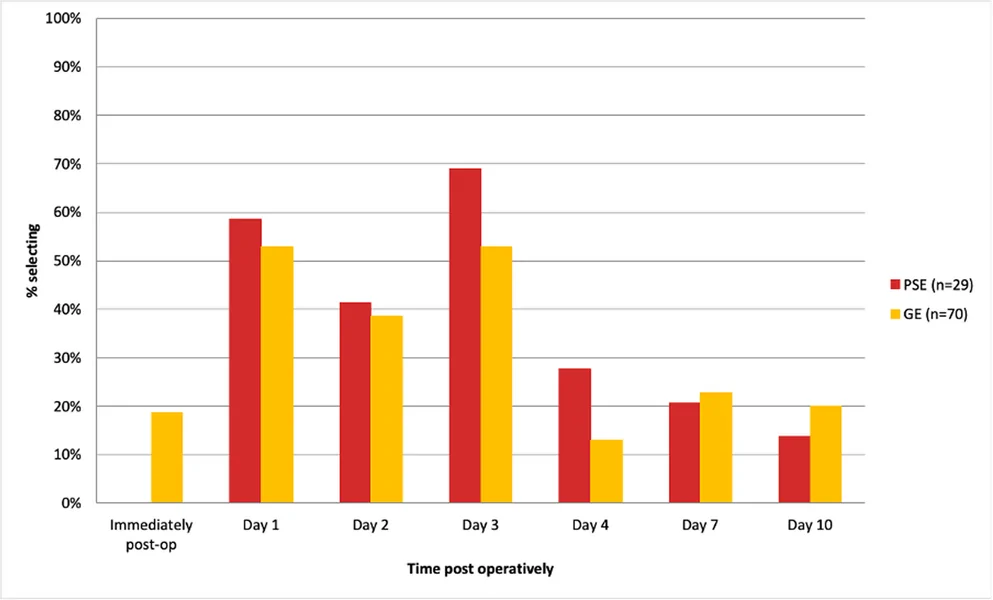
Timing of post-operative cortisol monitoring selected by pituitary subspecialty endocrinologists and general endocrinologists. Responses were multi-select, reflecting that clinicians may monitor cortisol at multiple timepoints. PSE were significantly less likely to select immediate post-operative monitoring than GE (0% vs 19%, p=0.009). Abbreviations: *PSE*, Pituitary subspecialty endocrinologist; *GE*, General endocrinologist;* Post-op*, post-operatively

Post-operative cortisol monitoring practice was assessed among endocrinologists only. Responses were multi-select, reflecting that clinicians may monitor cortisol at multiple timepoints (Fig. 3). Day 1 and day 3 were the most frequently selected monitoring timepoints (54/99, 55% and 57/99, 58% respectively), followed by day 2 (39/99, 40%), day 10 (18/99, 18%), day 7 (22/99, 22%), day 4 (17/99, 17%), and immediately post-operatively (13/99, 13%). PSE were significantly less likely to select immediate post-operative monitoring than GE (0% vs. 19%, *p* = 0.009). No significant differences between PSE and GE were identified for any other monitoring timepoint.

Post-operative MRI timing differed significantly between surgeons and endocrinologists (*p* = 0.0003). The majority of surgeons selected 3-month imaging (19/31, 61%), with a minority selecting imaging within 4 days (7/31, 23%). In contrast, 32/99 endocrinologists (32%) indicated they would defer post-operative MRI to another specialty, and a further 18/99 (18%) selected a 6-12-month interval. No significant difference in post-operative MRI timing was identified between PSE and GE.

There was no conformity regarding the timing of post-operative visual field assessment: 3 months was the most commonly selected interval overall (50/130, 39%), followed by 4–6 weeks (34/130, 26%), deferral to another specialty (28/130, 22%), 6–12 months (13/130, 10%), and within 4 days (5/130, 4%). No significant differences were noted between surgeons and endocrinologists or between PSE and GE.

Management of asymptomatic residual cavernous sinus disease demonstrated the strongest consensus of any question in the survey. Active surveillance was selected by 117/130 respondents (90%) with no significant difference between any group. Radiotherapy was only selected by 9/130 respondents (7%) and re-operation by 4/130 (3%).

### Radiation oncology practice

Radiation oncologists were asked about their preference between stereotactic radiosurgery (SRS) versus conventionally fractionated radiotherapy (CFRT) for a 1.5 cm³ recurrent tumour 4 mm from the optic apparatus which would fit technical criteria for SRS. SRS was the preferred mode of treatment radiotherapy by 60% of radiation oncologist respondents (9/15), with 6/9 clinicians delivering it as a single fraction SRS. The recommended dose prescription for SRS was interrogated by a free text box. In keeping with typical practice, the question of the total dose prescription was answered commonly as a range of total dose prescriptions (e.g. 14–16 Gy) rather than a fixed single dose, with a dose prescription envelope appropriate for the technical platform used (i.e. 50–80%). A third of these clinicians (3/9) preferred an approach with fractionated SRS in this scenario. They uniformly considered a dose of 25 Gy in 5 fractions to the 70–80% isodose envelope. When presented with a clinical scenario not suitable for primary SRS (large adenoma with rapid post-operative regrowth and contact with the chiasm), the most commonly recommended fractionation and dosing schedule was 50.4 Gy in 28 fractions (11/15, 73%), followed by 54 Gy in 30 fractions (2/15, 13%), with 45 Gy in 25 fractions and 50 Gy in 25 fractions each selected by one respondent (7%) (Table 2).

**Table 2:** Radiation oncologist responses to radiotherapy planning scenarios (*n* = 15)

Respondent	Scenario A	Scenario B			
	Recommended dose schedule	Modality preference	SRS dose	Fractions	Isodose prescription
1	50.4 Gy / 28#	SRS	14–16 Gy	1	80%
2	50 Gy / 25#	SRS	25 Gy	5	80%
3	50.4 Gy / 28#	SRS	12–15 Gy	1	50%
4	50.4 Gy / 28#	SRS	Dose depends on constraints	1	Variable
5	50.4 Gy / 28#	SRS	25 Gy	5	80%
6	50.4 Gy / 28#	Fractionated RT	—	—	—
7	54 Gy / 30#	SRS	16 Gy	1	Variable
8	50.4 Gy / 28#	Fractionated RT	—	—	—
9	50.4 Gy / 28#	Fractionated RT	—	—	—
10	50.4 Gy / 28#	Fractionated RT	—	—	—
11	54 Gy / 30#	SRS	25 Gy	5	70%
12	50.4 Gy / 28#	SRS	16 Gy	1	50% or 80%
13	50.4 Gy / 28#	Fractionated RT	—	—	—
14	45 Gy / 25#	SRS	16 Gy	1	80%
15	50.4 Gy / 28#	Fractionated RT	—	—	—

Scenario A: 70-year-old female, rapid regrowth post-resection, MDT consensus for fractionated RT

Scenario B: 45-year-old female, recurrent NFPA, 1.5 cm³, 4 mm from optic apparatus

Abbreviations: *RT,* radiotherapy; *SRS*, stereotactic radiosurgery. Dashes indicate no responses supplied

Full scenario stems are provided in the supplementary material

## Discussion

This is the first survey study on the clinical care of patients with NFPAs. Our large multidisciplinary dataset of 145 respondents across two countries demonstrates typical clinician decision-making in current practice and lays out future directions for research and guideline development. Overall, practice patterns demonstrated a relatively balanced distribution of concordance and heterogeneity, with agreement concentrated in major management decisions and variability observed predominantly in investigation, follow-up, and multidisciplinary processes. This pattern suggests that clinicians converge where evidence or shared clinical norms are strongest but diverge in domains where guidance is limited or absent. This is of importance given the high prevalence of NFPAs in pituitary clinics globally and the lack of NFPA-specific guidelines to date.

**Fig. 4:**
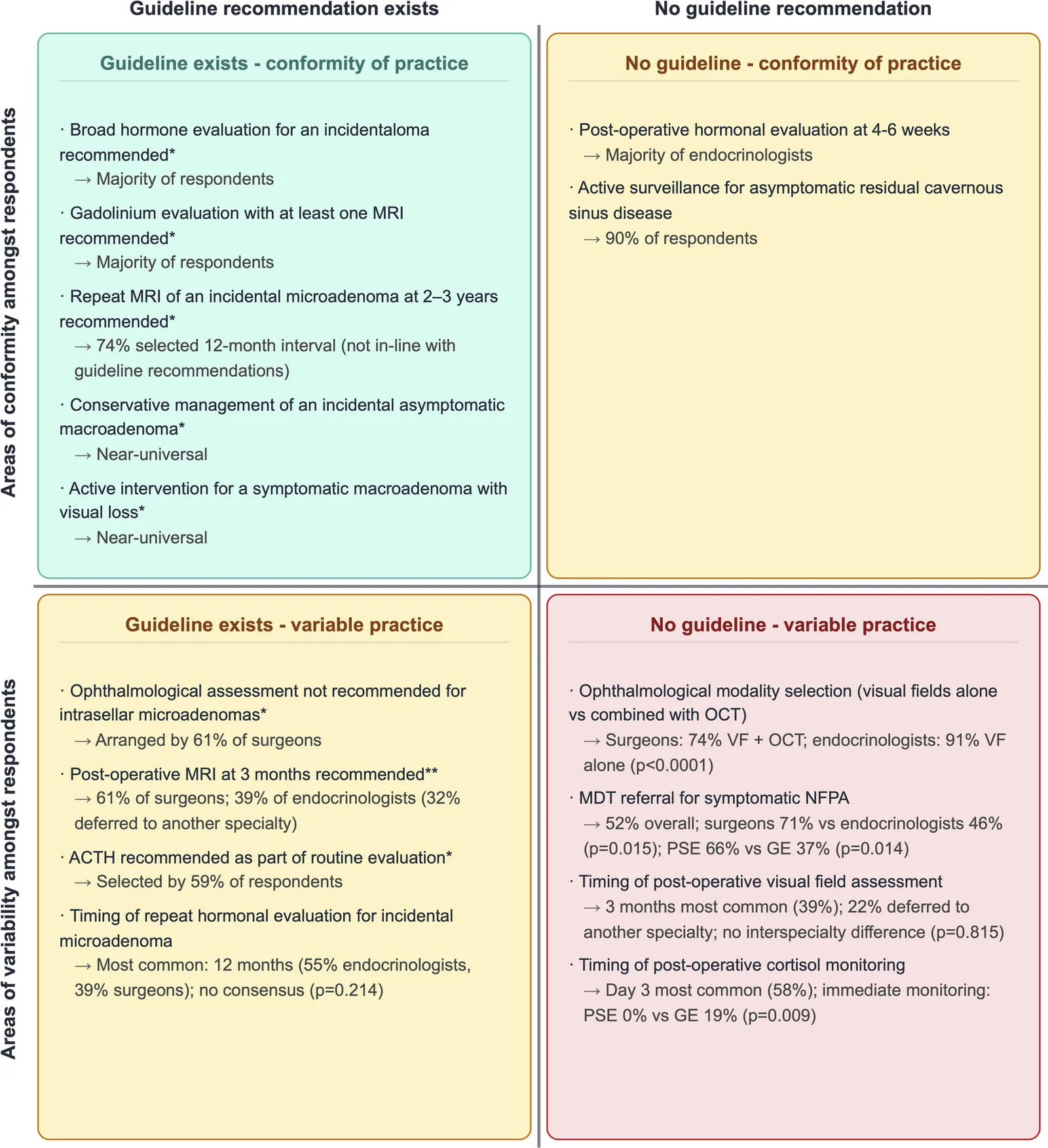
Matrix summarising areas of practice conformity and variability in the management of non-functioning pituitary adenomas, stratified by the availability of guideline recommendations. Abbreviations: *VF, *visual fields; *OCT,* optical coherence tomography; *ACTH,* adrenocorticotropic hormone; *NFPA, *non-functioning pituitary adenoma; *MRI,* magnetic resonance imaging; *MDT,* multidisciplinary team; *PSE,* pituitary subspecialty endocrinologist; *GE*, general endocrinologist. * Fleseriu M, Gurnell M, McCormack A, et al. Pituitary incidentaloma: a Pituitary Society international consensus guideline statement. Nat Rev Endocrinol. 2025;21(10):638–55. ** Ziu M, Dunn IF, Hess C, et al. Congress of Neurological Surgeons Systematic Review and Evidence-Based Guideline on Posttreatment Follow-up Evaluation of Patients With Nonfunctioning Pituitary Adenomas. Neurosurgery. 2016;79(4):E541–3.

Across the three clinical scenarios in the survey, we identified areas of both practice conformity and substantial heterogeneity spanning all aspects of care (pre- and post-operative clinical, hormonal, radiological, ophthalmological evaluation and management). The recent 2025 Pituitary Society incidentaloma guidelines [13] provide a framework against which several of our findings can be assessed, though notable gaps remain, particularly for the symptomatic NFPA presentation. In the discussion that follows, findings are contextualised against the limited available guideline recommendations, and areas without governing guidelines are explicitly identified to highlight where evidence gaps and future research is most needed (Fig. 4).

### Areas of conformity

The pattern of hormone evaluation performed by the majority of respondents (excluding radiation oncologists) included prolactin (92%), IGF-1 (90%), cortisol (89%), free T4 (89%), TSH (88%), LH (85%), FSH (82%), and androgens for men (79%). This pattern is broadly concordant with the 2025 Pituitary Society consensus recommendations for anterior pituitary evaluation in all patients with pituitary adenomas. Baseline pituitary hormonal deficiency is less common in microNFPA (< 1 cm) compared to macroNFPA (≥ 1 cm) but still has a prevalence of approximately 10% for any axis at the time of microNFPA diagnosis [14–16]. Notably, ACTH was selected by only 59%, discordant with consensus recommendations to include this as part of routine evaluation. This likely reflects known methodological challenges with ACTH measurement including assay instability, pre-analytical variability, and the need for specific collection conditions [17, 18].

Ophthalmological assessment for an intrasellar microadenoma was arranged by a minority of endocrinologists (21/99, 21%), with the majority aligning with the 2025 Pituitary Society consensus recommendation advocating against routine visual field testing in the microadenoma setting [13]. In contrast, the majority of surgeons arranged formal ophthalmological assessment (19/31, 61%), with 74% of this group selecting combined visual field perimetry and OCT. Recent evidence from the UK NFPA consortium reported that 1/459 (0.2%) patients with microadenomas required surgery due to visual compromise, corroborating smaller population studies reporting low rates of visual disturbance in microNFPA [15, 16, 19, 20]. The discrepancy between our survey dataset, these studies and the recent guidelines likely reflects entrenched clinical practice patterns as well as medicolegal caution.

Repeat MRI for an incidental microNFPA at 12 months was selected by 74% of all respondents. This is concordant with prior Endocrine Society guideline recommendations [21] but contrasts against the 2025 Pituitary Society consensus recommendation for a 2–3 year interval in the absence of deterioration in the interim. A recent study of 371 microNFPA patients followed for a median follow-up of 4.8 years demonstrated growth in 35% individuals; however, this was a median of 1 mm total growth and was not associated with clinical consequences [20]. Gadolinium contrast was used by 71% of surgeons and 65% of endocrinologists and is recommended for at least one study to best characterise pituitary lesions, acknowledging concerns regarding gadolinium deposition raised with repeated doses [22–24].

Conservative management of the incidental asymptomatic intrasellar macroadenoma in scenario 2 was adopted by 94% of all respondents, concordant with the 2025 Pituitary Society consensus and supported by evidence that growth is generally slow if present and not clinically significant within 6–12 months of initial diagnosis [25, 26].

Active intervention was equally near-universal in the setting of scenario 3 (a 58-year-old with visual deficits from a macroadenoma), as was repeat hormone evaluation at 4–6 weeks post-operatively amongst endocrinologists. Active surveillance for asymptomatic residual cavernous sinus disease was selected by 90% of all respondents, consistent with observational data demonstrating 90% freedom from regrowth at 6 years without routine adjuvant radiotherapy and evidence that long-term outcomes of adjuvant radiotherapy are not superior to salvage radiotherapy [27, 28].

### Areas of heterogeneity

The modality of ophthalmological assessment differed significantly between specialties, with most surgeons selecting combined visual field perimetry and OCT (74%) while most endocrinologists arranged visual field perimetry alone (91%, *p* < 0.0001). This likely reflects the absence of consensus recommendations regarding modality selection in this setting, longstanding local practices and differences in test access [13].

No consensus emerged regarding the optimal interval for repeat pituitary hormonal evaluation in the incidental microadenoma scenario, with the most common single answer selected by only 55%. The 2025 Pituitary Society consensus recommends repeat hormonal evaluation at 1–2 years in all patients with microadenomas and macroadenomas, or earlier in the presence of new clinical symptoms [13]. Hormone deficits are less commonly encountered in microadenomas than in macroadenomas [29, 30], with individual axis deficiencies present in fewer than 8% of patients at the time of microNFPA detection [16], and the risk of developing new endocrinopathies during follow-up is low and not significantly different between microadenomas and macroadenomas (0.9 versus 2.1 per 100 person-years, *p* = 0.15) [31]. Importantly, clinical symptoms and adenoma size do not always predict worsening endocrine function [15, 32, 33], supporting the rationale for interval hormonal re-evaluation regardless of imaging stability.

In the scenario of a symptomatic macroNFPA – a domain for which currently no multidisciplinary guideline exists – upfront pituitary MDT referral following clinical/radiological diagnosis was selected by only 52% of all respondents and differed significantly between surgeons and endocrinologists (71% vs. 46%, *p* = 0.015) and between PSE and GE (66% vs. 37%, *p* = 0.014). Post-operative MRI timing also differed significantly between surgeons and endocrinologists (*p* = 0.0003), the most common interval selected by surgeons being 3-months post-operatively, in accordance with CNS/AANS guideline recommendations [11]. In the context of this survey’s symptomatic macroadenoma scenario, which included Knosp grade 2 cavernous sinus invasion, a 3-month post-operative MRI is especially valuable as it establishes whether gross total or subtotal resection was achieved and thereby guides surveillance frequency. Contemporary data demonstrate that residual tumour progression and true recurrence represent temporally distinct phenomena. Most residual growth occurs within the first 5 years post-operatively and the majority of recurrences after complete resection arise beyond 5 years, supporting risk-stratified surveillance based on post-operative residual status [34–36]. In the absence of any established guidelines, post-operative visual field assessment timing showed no significant interspecialty difference (*p* = 0.815), with 3-month assessment the most common choice but with low level consensus (39% of respondents). However, there is evidence of visual field and acuity recovery after chiasmal decompression following a biphasic pattern of rapid early improvement within the first weeks to months, followed by a slower delayed phase for up to 3 years. Based on this trajectory, ophthalmological review within the first 3 months after surgery, repeated at 12 and 24 months may be suitable to adequately document both phases of recovery as well as detect early deterioration that may signal recurrence or remnant progression [37, 38].

### Differences between pituitary subspecialty and general endocrinologists

Within the endocrinologist group, PSE were significantly more likely to refer a symptomatic macroadenoma for MDT meeting discussion (66% vs. 37%, *p* = 0.014) (Fig. 2), are more aware of their referring surgeon’s annual operative caseload (93% vs. 51%, *p* = 0.005) and less likely to select immediate post-operative cortisol monitoring (0% vs. 19%, *p* = 0.009) (Fig. 3). The higher rate of immediate post-operative cortisol monitoring among GE likely reflects less exposure to pituitary post-operative care; cortisol measured immediately following surgery may be confounded by the physiological stress response and time of day, limiting its utility in reliably assessing true HPA axis function at this timepoint. Whether these practice differences translate into differential patient outcomes remains unknown and warrants prospective study, noting that a caseload-patient outcome relationship is well-established with pituitary surgeons [39, 40]. These findings support consideration of more formalised referral pathways and concentration of care within higher-volume centres to promote consistent, high-quality management.

### Radiation oncology management options

Radiotherapy remains a highly effective therapeutic option for progressive and inoperable NFPA, with local control rates at 5 years of around 94% after SRS [41] and up to 97% after CFRT [42], with CRFT usually reserved for large tumours and tumours in direct proximity or involvement of critical organs at risk (OaR) where the prescribed target tumour dose would exceed the radiation dose tolerance for the relevant OaR (e.g. optic nerves and optic chiasm).

The near-uniform preference for 50.4 Gy in 28 fractions for the indication of fractionated radiotherapy (Table 2) is concordant with published practice and guideline recommendations for pituitary tumours [11]. It is noteworthy that when compared to 50 Gy in 25 fractions, 50.4 Gy in 28 fractions is biologically nearly indistinguishable with respect to the predicted long-term tumour control probability as well as the potential risk of radiotherapy-associated late side effects. However, a dose prescription of 50.4 Gy in 28 fractions requires 3 additional treatment days which may have a negative impact on both the patient and local health care resources. The relatively low participation rate of radiation oncologists in this survey (*n* = 15) limits the conclusions that can be drawn from these descriptive findings.

The preference for SRS is consistent with its established role in small-volume progressive and inoperable or recurrent disease with an adequate distance from a dose-limiting critical OaR (e.g. optic apparatus) [41]. The question of dose prescription in this scenario was given as an open text box, acknowledging the heterogeneity of dose prescriptions associated with the available SRS platforms and individual patient-specific parameters (which were not provided in detail in our scenarios), and thus varied more widely. However, only 60% (9/15) of radiation oncologist respondents indicated SRS as their preferred option, possibly reflecting pre-existing established local practices and/or lack of local or regional access to a dedicated CNS radiosurgical platform/service.

## Conclusion

This survey study of endocrinologists, neurosurgeons, ENT surgeons, and radiation oncologists identifies both areas of practice conformity and substantial heterogeneity in the management of NFPA. Overall, there was no clear predominance of either concordance or heterogeneity, with clinicians demonstrating consistent agreement in some core management decisions alongside marked variability in several other domains of care. Conformity was most evident where guideline recommendations exist, while heterogeneity was concentrated in domains lacking any dedicated guidance, particularly for post-surgical surveillance structure. Differences between pituitary subspecialty and general endocrinologists further highlight the potential influence of case volume and multidisciplinary integration on clinical decision-making and, potentially, patient outcomes.

Noting the disconnect between the high burden of NFPA in pituitary clinics and relative paucity of NFPA research groups compared to functioning pituitary adenomas, our findings underscore the pressing need for comprehensive NFPA-specific assessment and management guidelines. Development of these guidelines will require international and multidisciplinary input and consideration of the full spectrum of clinical presentations to create evidence-based and feasible structured care pathways to reduce unwarranted practice variation and optimise outcomes for patients with NFPAs.

## Supplementary Information

Below is the link to the electronic supplementary material.

Supplementary Material 1 (DOCX 19.4 KB)

## Data Availability

No datasets were generated or analysed during the current study.
