# Supplementary material for: Clinician decision-making in non-functioning pituitary adenomas: an Australian and New Zealand interdisciplinary survey study
Source: Pituitary. 2026 Jul 3;29(4):117. doi: 10.1007/s11102-026-01716-3 (PMC13331897; doi:10.1007/s11102-026-01716-3)
Supplement: Supplementary file 1 — Supplementary Material 1 (DOCX 19.4 KB) [file 11102_2026_1716_MOESM1_ESM.docx]

**Supplementary File 1**

**Survey Instrument: ANZ Non-Functioning Pituitary Adenoma Decision Making Survey**

**Note to reader**

This document presents a synthesised version of the survey instrument. The original survey was administered electronically via Microsoft Forms and included branching logic such that questions were tailored to each respondent’s specialty. Questions are presented here in consolidated form with specialty-specific routing noted where relevant. Question numbering runs consecutively throughout this document.

**Section 1: Respondent demographics**

*The following demographic questions were presented to all respondents. Response options for case volume differed by specialty as indicated.*

**1.** What is your specialty?

☐ Neurosurgeon

☐ Endocrinologist

☐ ENT surgeon

☐ Radiation oncologist

**2.** Which state or territory do you work in?

☐ Victoria

☐ New South Wales

☐ Queensland

☐ Western Australia

☐ South Australia

☐ Tasmania

☐ Northern Territory

☐ Australian Capital Territory

☐ New Zealand

**3.** Where do you predominantly practise?

☐ Metropolitan

☐ Regional / Rural

**4.** Do you regularly (i.e., at least monthly) attend a hospital-based pituitary or base of skull multidisciplinary team meeting?

☐ Yes

☐ No

**5.** Do you work in a pituitary MDT clinic (i.e., more than one specialty seeing patients together)?

☐ Yes

☐ No

**6.** How many pituitary tumour cases do you see? (Surgeons and radiation oncologists: per year; Endocrinologists: per month)

☐ < 5 per month (endocrinologists)

☐ ≥ 5 per month (endocrinologists)

☐ < 20 per year (surgeons / radiation oncologists)

☐ 21–50 per year

☐ > 50 per year

**Section 2: Clinical scenario 1 — Incidental intrasellar non-functioning microadenoma**

| **Clinical scenario**  *A 30-year-old individual had an MRI head after sport-related trauma which revealed a 6 mm intrasellar pituitary lesion. No visual field defects were identifiable on examination.* |
| --- |

**7.** What endocrine testing would you perform? (Select all that apply)

☐ Refer to an endocrinologist for endocrine testing

☐ Adrenocorticotropic hormone (ACTH)

☐ Cortisol

☐ Follicle-stimulating hormone (FSH)

☐ Luteinizing hormone (LH)

☐ Oestrogen (if female)

☐ Progesterone (if female)

☐ Androgens (if male)

☐ Thyroid-stimulating hormone (TSH)

☐ Free T4

☐ Free T3

☐ Prolactin

☐ Insulin-like growth factor-1 (IGF-1)

☐ Growth hormone (GH)

**8.** What ophthalmological investigations would you arrange?

☐ None

☐ Visual field perimetry

☐ Optical coherence tomography (OCT)

☐ Visual field perimetry and OCT

**9.** Endocrine evaluation confirms the patient is eupituitary. When would you retest pituitary function?

☐ 1 month

☐ 3–6 months

☐ 12 months

☐ 2 years

☐ Discharge to GP with instructions

☐ No further retesting required

**10.** When would you arrange a repeat MRI scan?

☐ No repeat imaging

☐ 1 month

☐ 3–6 months

☐ 12 months

☐ 2+ years

**11.** Would you request gadolinium contrast for the repeat scan?

☐ Yes

☐ No

☐ N/A — no repeat imaging required

**Section 3: Clinical scenario 2 — Incidental intrasellar non-functioning macroadenoma**

| **Clinical scenario**  *A 70-year-old man had a fall at home and had neuroimaging performed. An MRI confirmed the presence of a pituitary macroadenoma with maximal dimension of 15 mm and unilateral cavernous sinus invasion (Knosp grade 2) without suprasellar extension. He underwent full hormonal evaluation and was eupituitary. Visual fields were normal on examination.* |
| --- |

**12.** What management do you institute? (Select one)

☐ Referral for formal visual field testing to assess need for surgery

☐ Repeat clinical, radiological and hormonal evaluation in 6 months

☐ Repeat clinical, radiological and hormonal evaluation in 12 months

☐ Immediate referral for surgery

☐ Immediate referral for radiotherapy

**Section 4: Clinical scenario 3 — Symptomatic non-functioning macroadenoma with visual compromise**

| **Clinical scenario**  *A 58-year-old male presented to his GP with concerns regarding his vision when driving at night. On confrontation there was a superior quadrantanopia and he was referred to your clinic. MRI brain confirmed a pituitary macroadenoma, 19 mm in maximal dimension, elevating the optic chiasm and invading the right cavernous sinus (Knosp grade 2). Serum prolactin was tested and was within the normal range.* |
| --- |

**13.** What are your next steps of management? (Select all that apply)

*Surgeons were presented with the option to proceed directly to surgery; endocrinologists were presented with the option to refer to a neurosurgeon.*

☐ Active surveillance with review in 6 months

☐ Full pituitary hormone evaluation

☐ Referral to an endocrinologist (surgeons) / Referral to a neurosurgeon (endocrinologists)

☐ Referral to ophthalmology

☐ Referral to a pituitary MDT

☐ Proceed directly to surgery (surgeons only)

☐ Refer to a radiation oncologist

**14.** If referring to a neurosurgeon, do you know the annual operative pituitary caseload of the surgeon to whom you most commonly refer? (Endocrinologists only)

☐ < 20 cases per year

☐ 21–50 cases per year

☐ > 50 cases per year

☐ Unsure

☐ N/A — not referred to neurosurgery

**Post-operative management**

*The following questions were presented after the scenario was extended: the patient underwent elective endoscopic transsphenoidal resection of the pituitary tumour with no intra-operative complications. Histopathology was consistent with a non-functioning pituitary adenoma.*

**15.** How long after surgery do you arrange the first repeat MRI?

☐ ≤ 4 days

☐ 4–6 weeks

☐ 3 months

☐ 6–12 months

☐ Defer to another specialty to arrange

**16.** When would you monitor early morning serum cortisol in the early post-operative period? (Select all that apply) (Endocrinologists only)

☐ Immediately post-operatively

☐ Day 1

☐ Day 2

☐ Day 3

☐ Day 4

☐ Day 7

☐ Day 10

**17.** How long after surgery do you arrange a repeat full pituitary hormone panel? (Select all that apply)

☐ Day 1 post-operatively

☐ Day 4 post-operatively

☐ 4–6 weeks post-operatively

☐ 3 months post-operatively

☐ 6–12 months post-operatively

☐ Defer to another specialty to arrange

**18.** How long after surgery do you arrange a repeat formal visual field assessment? (Select all that apply)

☐ ≤ 4 days

☐ 4–6 weeks

☐ 3 months

☐ 6–12 months

☐ Defer to another specialty to arrange

*Post-operative imaging revealed that the sella and suprasellar region were clear of tumour but there was residual tumour in the right cavernous sinus.*

**19.** How do you manage this residual disease?

☐ Active surveillance with serial MRI

☐ Refer to radiation oncology for radiotherapy of residual disease

☐ Plan for re-operation to resect the residual cavernous sinus disease

**Section 5: Radiation oncology scenarios (radiation oncologists only)**

*The following questions were presented to radiation oncologists only.*

| **Clinical scenario**  *Scenario A: A 70-year-old female underwent transsphenoidal resection of a large pituitary adenoma which impacted her vision. There is rapid regrowth over the next 2 years and a consensus decision is reached at a pituitary MDT meeting to offer fractionated external beam radiotherapy as the primary management option.* |
| --- |

**20.** What is your standard recommended fractionated radiotherapy dose schedule?

☐ 45 Gy in 25 fractions

☐ 50.4 Gy in 28 fractions

☐ 50 Gy in 25 fractions

☐ 54 Gy in 30 fractions

| **Clinical scenario**  *Scenario B: A 45-year-old female with a previously resected non-functioning pituitary adenoma presents to your clinic with recurrent disease measuring 1.5 cm³ (GTVp) and situated 4 mm from the optic chiasm and optic nerves.* |
| --- |

**21.** What form of radiotherapy would be your preferred approach?

☐ Stereotactic radiosurgery (SRS)

☐ Conventional fractionated conformal radiotherapy

**22.** What is your standard dose prescription in Gy?

**23.** Over how many fractions do you deliver the dose?

☐ 1 fraction

☐ 3 fractions

☐ 5 fractions

**23.** What is your standard isodose prescription envelope?

☐ 50%

☐ 70%

☐ 80%

☐ Other
